# Physiological indices and driving performance of drivers at tunnel entrances and exits: A simulated driving study

**DOI:** 10.1371/journal.pone.0243931

**Published:** 2020-12-17

**Authors:** Jinliang Xu, Xiaodong Zhang, Huan Liu, Kaige Yang, Fangchen Ma, Haoru Li, Yufei Sun

**Affiliations:** 1 School of Highway, Chang’an University, Xi’an, Shaanxi, China; 2 China Railway First Survey and Design Institute Group Company Limited, Xi'an, Shaanxi, China; Tongii University, CHINA

## Abstract

The entrance and exit sections of a tunnel are the accident black-spots in an expressway. For a safe operation of road tunnels, it is necessary to understand a driver’s physiological indices and driving performance when driving through tunnels. In this study, the UC-Win/Road simulation software was used to build 12 tunnel models of different lengths. A simulated driving experiment was carried out in a 6-DoF motion platform. The lateral position of vehicles characterizing the driving performance was measured using the motion platform. Electrocardiogram and eye movement data of 25 recruited drivers were collected simultaneously through the experiment. The spatial changes in a driver’s heart rate (HR) growth rate, RMSSD, pupil diameter growth rate and vehicle lateral deviation within 300 m before and after the tunnel entrance and exit were analyzed to determine the variation rules in the different tunnels. The study identified the length range in the tunnel entrance and exit sections that influences the drivers. A quantitative analysis was further carried out to analyze the relationship between the physiological indices and the driving performance indicator. The results showed that a driver’s heart rate fluctuates significantly 250 m before the tunnel entrance and 50 m before the exit. In this region, the pupil diameter increases gradually, and drivers tend to shift the vehicle to the left. At the tunnel exit, the HR and RMSSD are affected significantly by the tunnel length, and the variation is higher in longer tunnels. In comparison, the tunnel length has no significant effect on the physiological indicators and driving performance of the drivers at the entrance and exit.

## 1. Introduction

One of the main causes of traffic accidents in tunnel entrance and exit sections is the significant variation in the psychophysiological response and behavior of drivers when entering and leaving the tunnel [1–3]. Thus far, ample studies have been conducted on the physiological indices and driving performance of drivers in tunnels. Fu et al. concluded that in-car navigation has a significant influence on a driver’s perceptual response and driving behavior at tunnel entrances, and the influence degree was different in different regions [4]. By collecting eye movements (number of fixations, duration of fixations, and number of saccades) and driving performance (speed, steering wheel, and vehicle lateral position), Wang et al. reported a gradual increase in the number of fixations, duration of fixations and number of saccades in the transition zone. Moreover, the drivers were more cautious when driving in the tunnel entrance section as they tend to drive at lower speeds while decreasing the steering wheel and vehicle lateral position [5]. Kircher et al. conducted driving simulation experiments to study the influences of tunnel wall color and lighting on drivers’ visual attention and driving behavior, and the results showed that light-colored tunnel walls can aid drivers’ visual attention on the front than strong lighting [6]. Multivariate and univariate analyses of variance were applied to analyze drivers’ heart rate (HR) and driving performance at the tunnel entrance, inside the tunnel, and in the open road. The results showed that the stress level on drivers at the tunnel entrance was the highest, and drivers tend to decelerate before entering the tunnel and accelerate before exiting [7]. Through simulation experiments, tunnel models with different luminance levels were established to study the effects of luminance on the visual perception and speed control. Speed overestimation was reduced, and the reaction time of drivers was shorter under higher luminance; the lower the speed overestimation, the shorter the response time [8]. Yang studied the physiological and behavioral characteristics of drivers at the tunnel entrance, and built a regression model under speed limits of 80 km/h and 60 km/h considering the speed and HR growth rate [9]. The HR growth rate is defined as the percentage of HR increment between active and rest periods. Luo et al. established a regression model of the luminance, vehicle speed, and HR growth rate in the case of mountainous tunnels, focusing on the relationship between drivers’ workload, luminance, and vehicle speed [10]. The above research focused on the influence of tunnel entrances and exits on both drivers’ physiological indices and driving behavior through field or simulation experiments [4–10]. Some scholars only studied the influence of tunnels on the driving performance. Calvi and D’Amico studied the behavior of drivers while approaching a tunnel, inside the tunnel, and leaving the tunnel by conducting a simulated driving experiment. It was concluded that the vehicle lateral position (LP) was lower, and the speed was lower in the tunnel than in the open road section [11]. Using a combination of simulator experiment and field experiment, Akamatsu et al. studied the impact of tunnels on the driving behavior. The results showed that the accelerator stroke decreases when a vehicle enters or leaves the tunnel. The study also proved the effectiveness of a driving simulation in understanding a driver’s perception of the road structure and driving behavior [12]. Tornos also adopted field experiments and driving simulation experiments. A comparison of the field experiment and relevant simulation showed that the driving speed in the simulated tunnel is higher than that in the actual tunnel. Moreover, the LP remained unchanged in the straight segment; however, the LP deviation in the simulated tunnel was significant while driving on a curve with a radius of 375 m [13]. The vehicle speed, LP, and subjective workload were collected through simulation experiments. Shimojo et al. found that the different types of tunnel cross-sections have no significant effect on the average vehicle speed and LP [14]. The above studies adopted a driving simulation method to study the changes in the vehicle speed, acceleration, and LP in tunnel sections. In addition, some researchers have studied the influence of tunnel environment on the drivers in terms of their physiological behavior. Based on a backpropagation (BP) neural network, Liu et al. established a mathematical model of the fixation duration, number of fixations, and saccade amplitude at the tunnel entrance. The results showed that the fixation duration increases gradually as the vehicle approaches the tunnel entrance, whereas the number of fixations and saccade amplitude decrease [15]. Du et al. used an EMR-8B eye tracking device to collect the pupil area of eight drivers in 26 typical tunnels. The analysis showed that the pupil area at the tunnel entrance follows a power function relationship with the pupil luminance, which varies drastically within a range of 10 m from the tunnel entrance [16]. A relationship model between the eye movement parameters and the distance was established by analyzing the experimental data of nine drivers at the tunnel entrance and inside the tunnel. Within a range of 100 m before and after the entrance, the eye movement parameters changed significantly [17]. Liu et al. and Ding et al. studied the variation rules of drivers’ visual characteristics at the tunnel entrance, and built relevant models [18,19]. They further studied the impact of driving environment by varying the tunnel length (short, long and extra-long tunnels) on drivers’ physiological indices, and concluded that the psychological indices vary in the different tunnel sections (entrance, driving, and exit sections) [20]. Qi et al. studied the spatial variation characteristics of the eye movement and electrocardiograph (ECG) in the tunnel environment, and found that a three-order spline interpolation curve could better reflect the dynamic changes in the average pupil diameter and HR growth rate [21].

Clearly, studies on the physiological and behavioral characteristics of drivers were conducted with a focus on tunnel entrances. Most current studies concentrated on visual, ECG, and behavioral characteristics of drivers under driving simulation conditions. To investigate the effects of tunnel environment on a driver’s physiological response and driving performance at tunnel entrances and exits, two main limitations need to be addressed. First, comparisons between tunnel entrances and exits have rarely been verified in existing studies. Second, few researchers correlated the physiological response with the driving performance. Consequently, it is impossible to explore the underlying mechanism of driving behavior variation in terms of the psychological condition.

In this study, visually immersive tunnel models with different lengths (1000–20000 m) were built to analyze the variations in the physiological indices and driving performance at the tunnel entrance and exit. The two main advantages of this study are as follows. First, the spatial distributions of the physiological indices and driving performance are clearly indicated. Second, the results specify the regions in the tunnel entrance and exit sections where the physiological indices and driving performance are strongly influenced. The findings are promising for highway design with a focus on improving tunnel safety.

The rest of this paper is organized as follows. The simulation experiment and data processing method are presented in section 2. The subsequent section describes the spatial distributions of the physiological indices and driving performance, and presents a comparison between the tunnel entrance and exit sections. Our research findings are summarized in the context of previous studies in the “Discussion” section. Finally, the conclusions are drawn at the end.

## 2. Materials and methods

### 2.1. Zone division at tunnel entrance and exit

**Fig 1:**
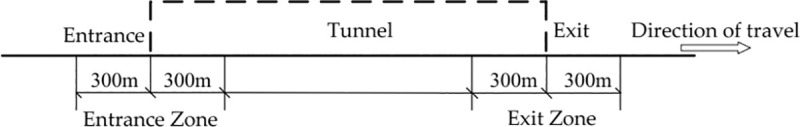
Zone divisions at tunnel entrance and exit.

In this study, traffic accident characteristics of a tunnel section are considered to divide the tunnel entrance and exit sections. Amundsen and Ranes studied traffic accidents that occurred in several tunnels in Norway, and showed that the accident rate is the highest within 100 m of the tunnel entrance and exit [22]. Lemke analyzed the accident data of German autobahns and tunnels, and found that tunnel accidents mainly occurred at the entrance [23]. Yeung and Wong analyzed the characteristics of 608 road tunnel traffic accidents in Singapore from 2009 to 2011, and found that the accident rate 250 m before and after the entrance of the tunnel was significantly higher than those in the other sections [24]. Lai et al. analyzed 2,193 traffic accidents in China, and found that the number of traffic accidents that occurred within 200 m of the tunnel entrance and exit accounted for most of the total traffic accidents in the tunnel section [25]. The above research shows that the accident rate strikingly increases at a certain distance from the tunnel entrance and exit. The value of this distance varies from 100 m to 250 m in the different studies. Given that a driver’s physiological responses and driving behavior may have a certain time difference, a distance of 300 m inside and outside the entrance and exit is selected as the length of the tunnel entrance and tunnel exit sections, as shown in Fig 1.

### 2.2. Driving simulation experiment

To study the physiological indices and driving performance of drivers at the tunnel entrance and exit, large amounts of vehicle driving data and physiological data are necessary. Considering the risk of field experiments, simulation experiments are often performed in relevant research, though data obtained from field experiments are more convincing [26,27]. The development of modern technology provides a technical support. A simulator has been validated as a useful research tool for evaluating visually perceived road structures [28]. Therefore, in this study, a driving simulator was used to assess the drivers’ physiological state and driving performance considering safety and repeatability of the experiments.

#### 2.2.1. Participants

The test drivers were recruited through an advertisement posted on the bulletin board of Chang’an University and WeChat from May 20, 2020 to May 25, 2020. All participants were ensured to be in good health and the uncorrected visual acuity was greater than 5.0 to reduce the individual differences in physiological response and performance. Meanwhile, all selected participants had to fulfil the requirements, which are to hold a driver’s license for at least 5 years (M = 10.6, SD = 2.9) and must have driven for at least 20,000 km during the last year. A total of 25 paid drivers took part in the study. The participants were aged from 30 years to 55 years (M = 42, SD = 6.8) and comprised 17 men (68%) and eight women (32%), which reflect the overall gender and age distribution of drivers in China. We mainly concentrated on their physiological indices and driving performance in the tunnel sections. The recruited participants had experience driving through tunnels. Table 1 lists the basic information of the participants, who were informed of the general conditions of the experiment and signed an informed consent (S1 Text). The research was reviewed and approved by Chang’an University before the experiment (S2 Text). The research content strictly follows the Declaration of Helsinki. The electrocardiosignal and eye movements of the drivers were collected during the experiments. The conductive paste used in the experiment had no side effects on the skin, and the SMI wireless glass used had no side effects on the eyes.

**Table 1:** Basic information of the participants.

Number	Gender	Age	Driving year	Occupation
1	Male	53	12	Teacher
2	Female	51	14	Corporate employee
3	Male	48	10	Merchant
4	Male	52	12	Teacher
5	Female	36	8	Corporate employee
6	Male	38	9	Taxi driver
7	Male	35	8	Corporate employee
8	Female	40	12	Teacher
9	Male	35	10	Taxi driver
10	Male	38	7	Civil servant
11	Male	39	7	Corporate employee
12	Female	48	15	Teacher
13	Male	55	16	Teacher
14	Male	34	7	Seller
15	Female	34	8	Teacher
16	Female	30	7	Taxi driver
17	Male	36	10	Corporate employee
18	Male	47	13	Merchant
19	Male	41	12	Teacher
20	Female	45	11	Teacher
21	Male	49	16	Taxi driver
22	Male	43	8	Teacher
23	Male	36	9	Merchant
24	Female	47	15	Corporate employee
25	Male	43	8	Taxi driver

#### 2.2.2. Driving simulation environment

**Fig 2:**
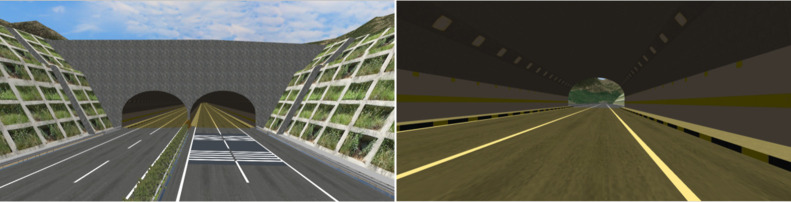
Simulated tunnel environment. a) Tunnel entrance. b) Tunnel exit.

A comprehensive survey of existing tunnels was conducted to make the simulated environment highly consistent with the real environment. The length of the studied tunnels ranged from 1000 m to 20000 m. Two assumptions were made: First, drivers could see the exit from the entrance in the short tunnels, and the driving time is shorter inside in the short and medium tunnels. Second, we focused on the changes in the drivers’ physiological indices and driving performance within a 300 m range of the tunnel entrance and exit. Therefore, short and medium tunnels were not included in this study. The tunnel lengths were designed to be 1010, 1510, 2010, 2520, 3018, 4019, 5033, 6000, 8000, 10000, 15000, and 20000 m. For the simulated models, the geometric features, road facilities, and road environment were consistent except for the tunnel length, to avoid the influence of these factors on the experimental results. The UC-win/Road modeling software was then used to build the simulation models (Fig 2). The mainline is a typical bidirectional four-lane-divided freeway with a design speed of 120 km/h and, the speed limit in the tunnel is 80 km/h. To avoid the influence of road environment other than the tunnel on the experiment as much as possible, shrub vegetation with a height of 1.5 m and an interval of 2 m were set in the median strip of the expressway, and the height of the subgrade filling and excavation was less than 5 m in all the simulated scenes. The lighting within the tunnel was designed strictly in adherence to the Chinese Guidelines for Design of Lighting of Highway Tunnels. The light condition was adjusted by varying the display brightness. The image brightness was adjusted at distances less than 100 m to simulate the effect of vision adaptation when entering a tunnel. The ceiling of the tunnels was dark, with lamps mounted in rows in the driving direction. The traffic signs and markings were designed in adherence to the Chinese Specification for Layout of Highway Traffic Signs and Markings. The signs of the speed limit, and tunnel length were set at the tunnel entrance. The lane lines inside the tunnel were set as white solid lines to prevent lane changes and overtaking. Tunnel portals and auxiliary facilities were designed by referring to actual roads to provide a realistic scene as much as possible. To eliminate the impact of alignment on the vehicle performance, the horizontal alignment was made tangent at the tunnel entrance and exit, and the vertical slope was set to 0.3%. In addition, a sunny weather and no other traffic flow were set to exclude the influence of the weather and traffic flow on the experiment.

#### 2.2.3. Experimental facilities

**Fig 3:**
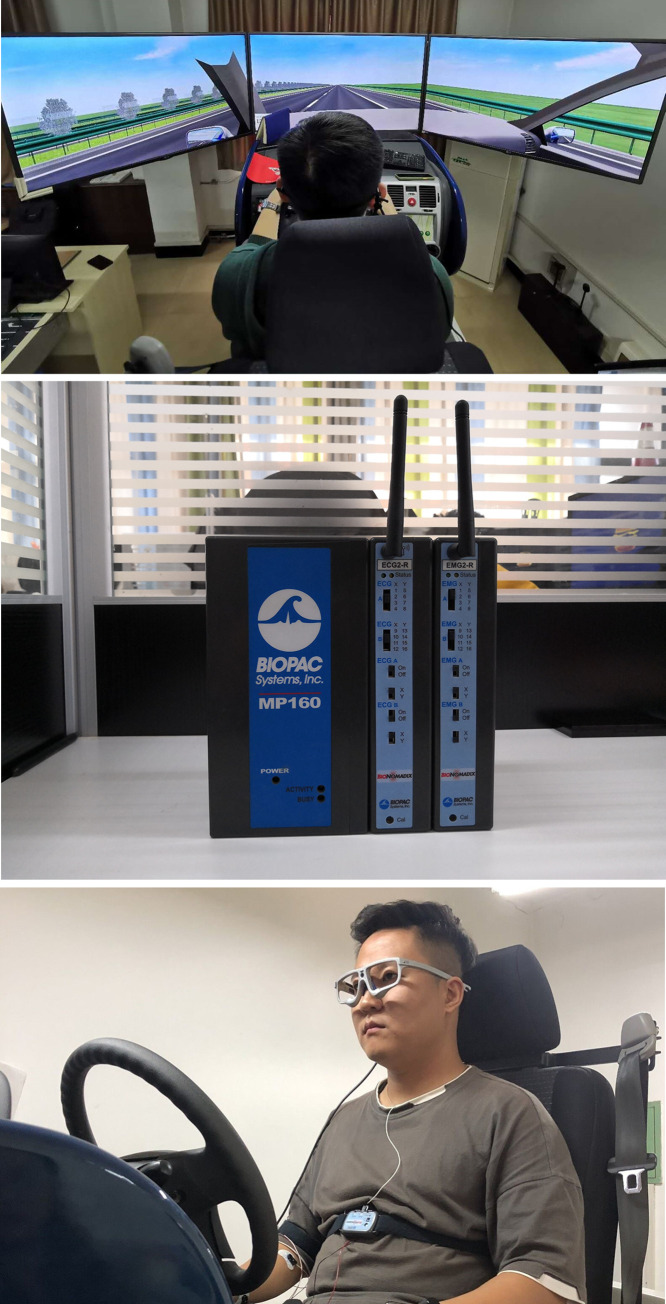
Experimental facilities. a) 6-DoF motion platform. b) MP150. c) SMI wireless glasses.

The main experimental facilities in this study were a 6-DoF motion platform, an MP150 multi-conductivity physiological recorder, and an SMI wireless glasses. The images and technical parameters of the experimental facilities are shown in Fig 3 and Table 2. The experimental system was also equipped with some auxiliary devices, such as a finger clip medical pulse meter, a medical patch electrode, and a stopwatch.

**Table 2:** Technical parameters of experimental facilities.

Facility	Technical parameter
6-DoF motion platform	The simulator system can provide a highly realistic virtual driving environment. The visual system provides a 130° horizontal and 40° vertical visual field in front. The sound system simulates the amplitude of the road and exhaust noises from other vehicles. The moving system provides the drivers with a sense of acceleration, deceleration, steering, and sideslip movement. The driving simulator with its own sensors can record real-time vehicle motion data such as the speed, lateral offset and steering wheel angle.
MP150 multi-conductivity physiological recorder	The MP150 physiological recorder and ECG amplifier help collect and analyze continuous ECG data. These have many advantages including flexible portability, custom mode, and on-line processing.
SMI wireless glasses	SMI wireless glasses can be used to collect eye-movement data such as the blink rate and pupil diameter. It has an advanced eye-movement tracking technology to ensure that the system can collect high-precision eye-movement data; the device is easy to operate and flexible to use.

#### 2.2.4. Experimental procedure

**Fig 4:**
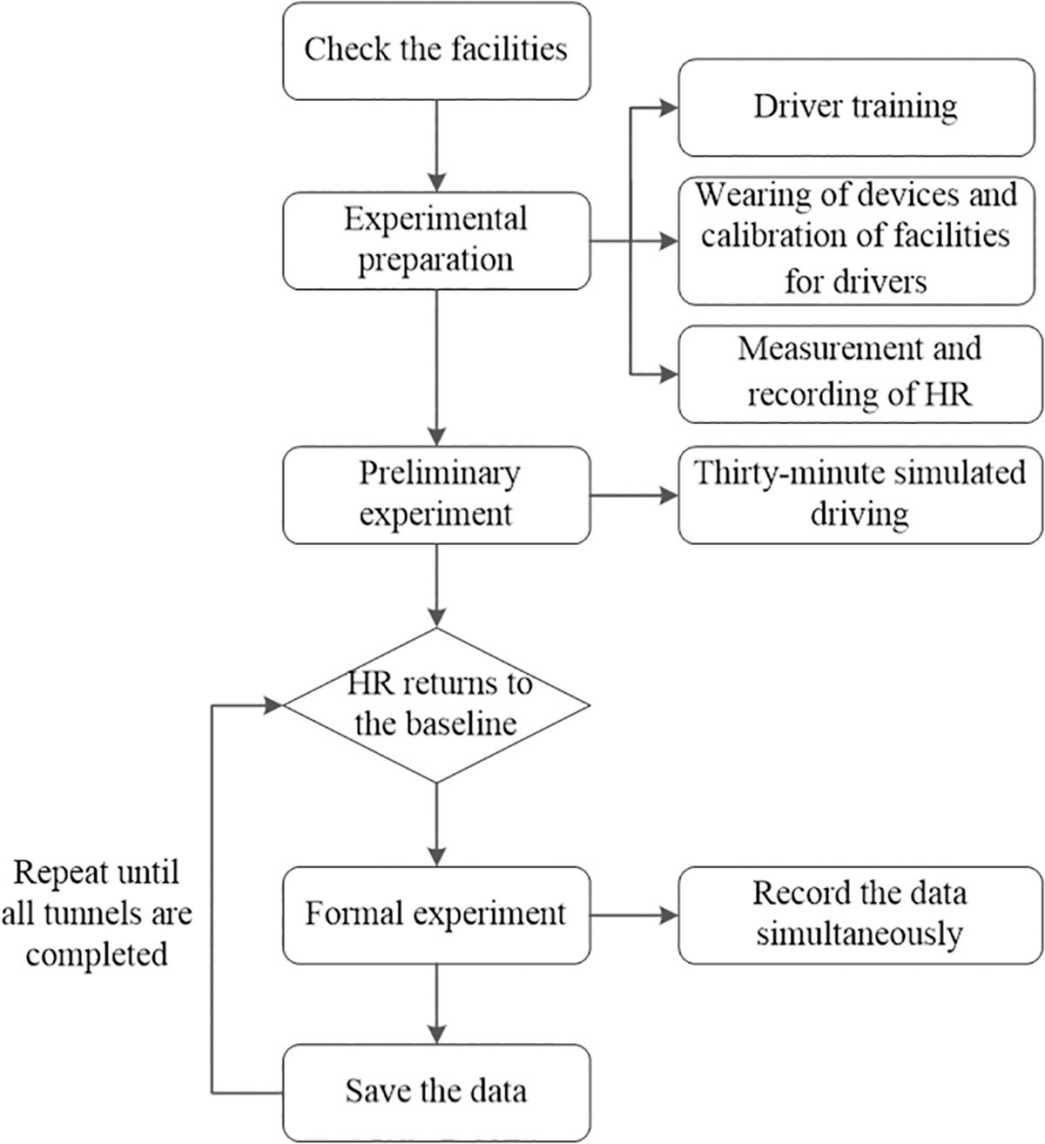
Experimental procedure.

The experiment in this study was carried out on the premise of driving safety. The entire simulation driving experiment comprised three stages: experimental preparation, preliminary experiment, and formal experiment. The specific experimental procedure is shown in Fig 4. The experiment was carried out at the road simulation laboratory of Chang’an University from June 10th to 30th, 2020. Before the experiment, the participants were asked to rest until their HR returned to the baseline. For each simulated scene, the participants took turns while performing the experiment to prevent fatigue. The experimental procedure was repeated until the simulation experiments in all the tunnel sections were completed.

### 2.3. Data processing

#### 2.3.1. Physiological indices

The light conditions vary significantly in the tunnel section, and visual perception has a significant influence on the drivers [29,30]. The eye movements can reflect the changes in the physiological state to a certain extent [5,15–17]. In addition, ECG indices have been widely used in studying the physiological responses of drivers in tunnel sections [31–36]. In this study, the HR growth rate, RMSSD, and pupil diameter growth rate were chosen as the characteristic indices reflecting the drivers’ physiological response in the tunnel sections. The HR and root mean square of the successive differences between adjacent N-N intervals (RMSSD) of the drivers can be directly derived from the physiological data analysis software AcqKnowledge5.0. Similarly, the pupil diameter can be extracted using Begaze3.7. Because of the individual differences between drivers, it is unadvisable to simply analyze the HR and pupil diameter values. To exclude the individual differences, we used the compensated values for the HR and pupil diameter to reflect the impact of tunnel environment on the driver. They are defined as the HR growth rate (GR_HR_) and pupil diameter growth rate (GR_PD_). The calculation formulae are expressed in (1), and (2).

$${GR}_{HR}=\frac{HR-{HR}_{basic}}{{HR}_{basic}}*100$$ (1)

$${GR}_{PD}=\frac{PD-{PD}_{basic}}{{PD}_{basic}}*100$$ (2)

where HR indicates the number of beats per minute; HR_basic_ indicates the heart rate of the driver in the rest state; PD indicates the driver’s pupil diameter at a certain moment, mm; and PD_basic_ indicates the pupil diameter of the driver in a normal environment, mm.

#### 2.3.2. Driving performance quantification

The main task of vehicle control is the LP control [37]. The measurement of a vehicle’s LP is a good method to evaluate the driving performance [38,39]. Impacted by the tunnel sidewall, lane keeping is particularly important in tunnels [40]. In this study, the LP was adopted to reflect the driving performance of the drivers. The original data of the vehicle LP can be obtained from the driving simulation platform through the UC/ WIN-Road software. The real LP can be calculated using Eq (3).

$$LP=|D_{RB}-D_{C-RB}|*100$$ (3)

where D_RB_ is the lateral distance of the vehicle with respect to the right boundary of the road derived from the driving simulation platform; D_C−RB_ is the distance between the center line of the driving lane and the right boundary of the road and is set to 3.9367 m based on the system setting.

## 3. Results

In exploring the physiological indices and driving performance at the tunnel entrance and exit, the spatial change rules of the HR growth rate, RMSSD, pupil diameter growth rate, and LP were first analyzed separately. The correlations between the physiological response and the driving performance were studied. Further, a comparison between the four indices in the tunnel entrance and exit zones was conducted.

### 3.1. Physiological indices and driving performance in tunnel entrance zone

**Fig 5:**
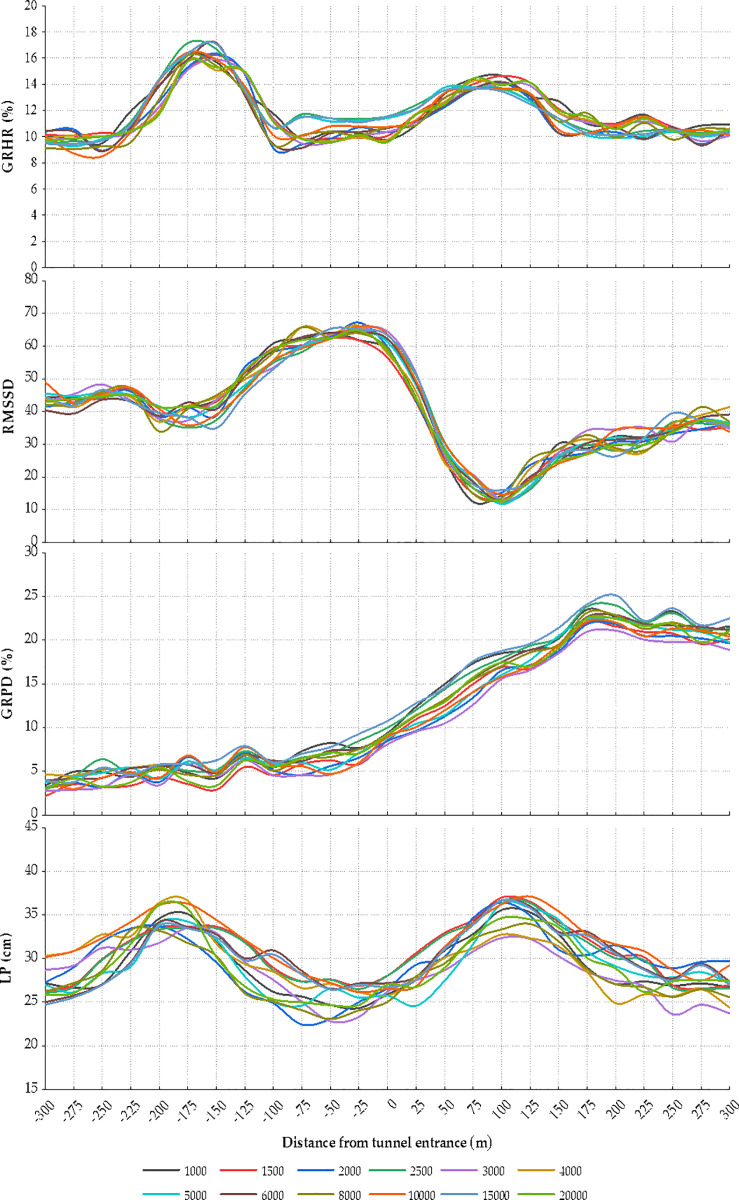
GRHR, RMSSD, GRPD, and LP at the tunnel entrance of different tunnels.

The analysis process was demonstrated by taking the HR growth rate as an example. Based on the data processing method in Section 2.3, the mean HR growth rate of the drivers was calculated. The distribution of the HR growth rate, RMSSD, the pupil diameter growth rate, and LP at tunnel entrances of different lengths is displayed in Fig 5. As shown, the HR growth rates of the drivers follow a similar change pattern. A statistical analysis method was used to quantitatively analyze the influence of the tunnel length on the HR growth rate. The overall HR growth rate does not follow the normal distribution (Sig. < 0.05). The Kruskal–Wallis test was then used to test the difference in the HR growth rate in the different tunnels. The test results show that there is no significant difference in the HR growth rates between the drivers in the different tunnel entrance sections. Therefore, the tunnel length has no effect on the HR growth rate. The maximum difference between the different tunnels is 2.63%, which may be due to an accidental error. As shown in Fig 5, the HR growth rate at the entrance, i.e., from 250 m before the tunnel to 200 m into the tunnel, shows an evident change, indicating that the drivers become nervous while approaching the tunnel environment. In the section ranging from 175 m to 250 m before the entrance, the HR growth rate increases significantly from 9.116% to 17.157%. The HR growth rate then decreases rapidly in the section ranging from 150 m to 100 m before the entrance and tends to stabilize thereafter. After entering the tunnel, the HR growth rate increases gradually and approaches a peak (13.104%–14.651%), from 75 m to 100 m after the entrance. Thereafter, the HR growth rate reduces until leveling off.

The same process was carried out to analyze the other three indices. The spatial changes in the RMSSD, pupil diameter growth rate, and LP show no significant difference (P < 0.05) at the entrances of the tunnels with different lengths. The change rules of the RMSSD and LP are consistent with that of HR growth rate. Evidently, the RMSSD fluctuates significantly in the section 225 m before the entrance and 200 m into the tunnel. The RMSSD 100 m into the tunnel is the lowest indicating that the heart rate variability is highest at this point. The vehicles turn left before entering the tunnel, showing a fluctuation in the LP. In the section 50 m before the entrance and 25 m into the tunnel, the LP maintains a minimum value. After entering the tunnel, the vehicles have a left-leaning tendency. It could be inferred that the drivers are more cautious when entering the tunnel while decreasing the LP. In addition, the vehicle is driven on the right side and the rudder is on the left in China. Under the influence of the right tunnel wall, the drivers may shift to the left within the lane. As shown in Fig 5, the change in the pupil diameter growth rate is significantly different from those of the HR growth rate and RMSSD. The pupil diameter growth rate exhibits an upward trend in the tunnel entrance zone until decreasing slightly from 175 m into the tunnel. It could be speculated that lighting is the major factor influencing the eye movement, and a certain time is required for the drivers to acclimatize to the tunnel environment.

### 3.2. Physiological indices and driving performance in tunnel exit zone

**Fig 6:**
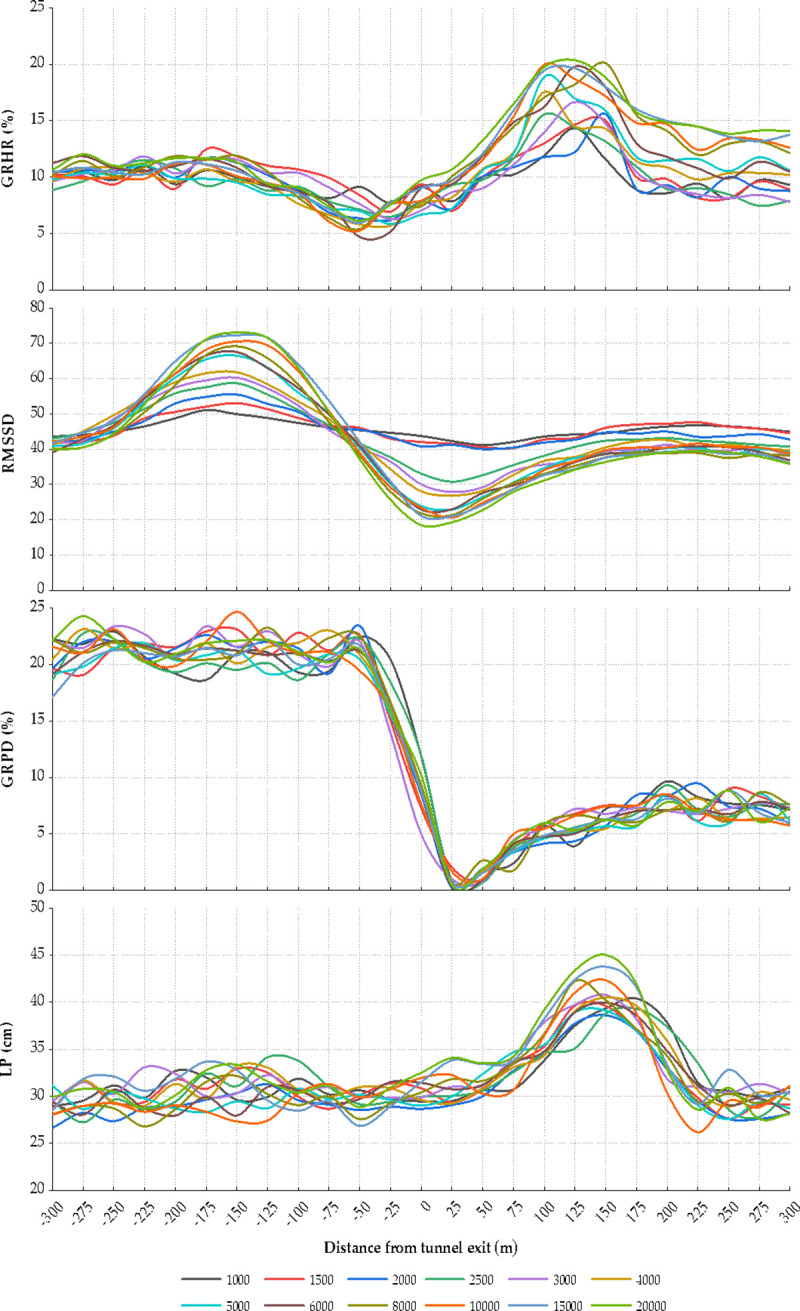
GRHR, RMSSD, GRPD, and LP at the tunnel exit of different tunnels.

The spatial distribution of the HR growth rate, RMSSD, pupil diameter growth rate, and LP are illustrated in Fig 6. Unlike in the entrance zone, the variations in the HR growth rate and RMSSD in the exit zone are consistent in the tunnels of different lengths; however, the variation range is quite different. A significance test of difference for the HR growth rate, RMSSD, pupil diameter growth rate, and LP was conducted. The data analysis confirms that the HR growth rate and RMSSD show marked differences (P > 0.05) between each tunnel, whereas the pupil diameter growth rate and LP show no significant difference (P < 0.05). The two indices are analyzed.

The test results of the HR growth rate are listed in Table 3. Taking the tunnel length of 4000–5000 m and 6000–8000 m as the boundary, the influence of tunnel exit environment on the HR growth rate can be divided into three subsets. The difference in the drivers’ HR growth rate between each subset is significant, and the influence gradually increases with the increase in the tunnel length. This may be attributed to the cumulative effect due to long-time driving, i.e., it is closely related to the significant increase in the tunnel length.

**Table 3:** Kruskal–Wallis test results of the HR growth rate at the tunnel exit.

Subset	Tunnel length (m)	F	Sig. (two-side test, α = 0.05)
1	1000–4000	14.703	0.100
2	5000, 6000	15.382	0.080
3	8000–20000	10.588	0.194

Similarly, the test result of the RMSSD is listed in Table 4. The influence of tunnel exit environment on the RMSSD can also be divided into three subsets with demarcations of 1000–2000, 2500–4000, and 5000–20000 m. The difference in the drivers’ RMSSD is higher in the case of longer tunnels. It could be speculated that the long-time driving increases the stress level of the drivers, thus fluctuating their heart rate.

**Table 4:** Kruskal–Wallis test result of the RMSSD at the tunnel exit.

Subset	Tunnel length (m)	F	Sig. (two-side test, α = 0.05)
1	1000–2000	14.661	0.140
2	2500–4000	13.685	0.257
3	5000–20000	10.635	0.133

**Fig 7:**
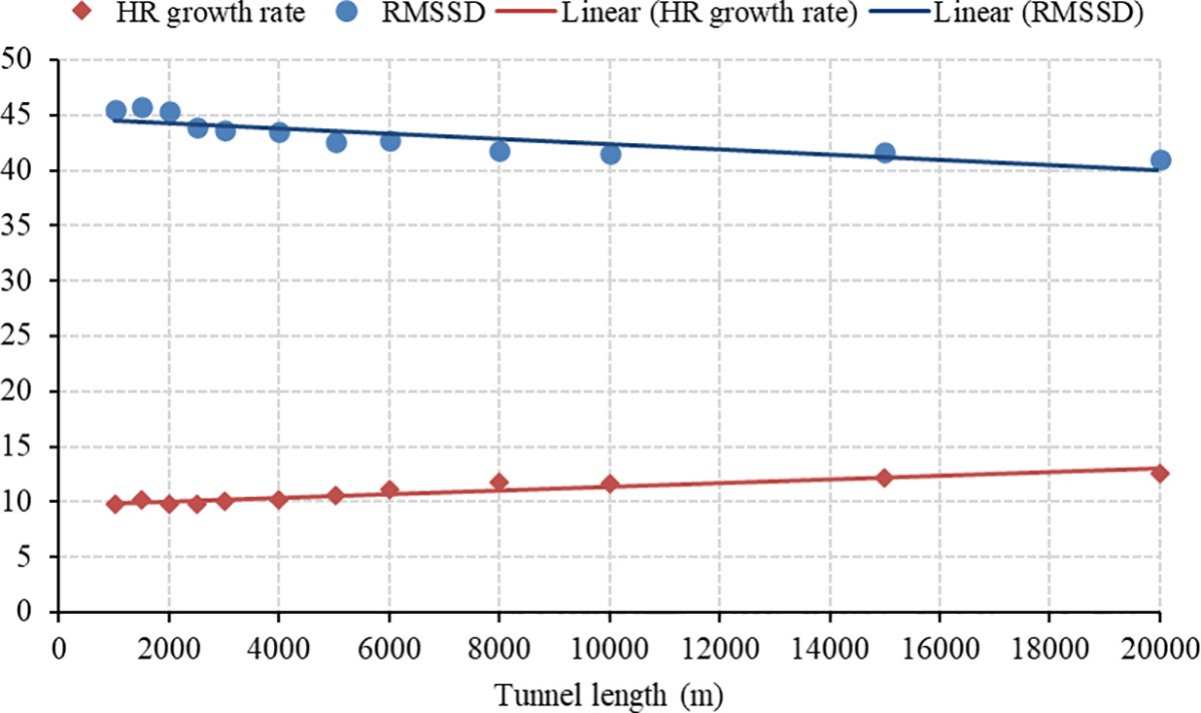
Regression models for the HR growth rate and RMSSD.

The SPSS software was used to fit the relationship between the mean HR growth rate, RMSSD, and tunnel length. The fitting parameters and models are shown in Table 5 and Fig 7.

**Table 5:** Fitting parameters.

Index	Regression	R^2^	Constant	b1
HR growth rate	Linear	0.704	44.779	-0.0002
RMSSD	Linear	0.877	9.7443	0.0002

The regression model between the HR growth rate and the tunnel length is expressed in Eq (4), and the regression model between the RMSSD and the tunnel length is expressed in Eq (5).

$${GR}_{HR}(L)=-0.0002L+44.779$$ (4)

RMSSD(L)=0.0002L+9.7443 (5)

It is important to note that Eqs (4) and (5) can only be used to calculate the HR growth rate and RMSSD for tunnel lengths in the range of [1000 m, 20000 m]. The HR growth rate increases monotonously whereas the RMSSD decreases monotonously with the increase in the tunnel length. It could be inferred that driving in longer tunnels increase a driver’s stress level. The driver may not handle potential emergencies timely under high-stress states, thus impairing the driving safety in longer tunnels.

The specific spatial distributions of the four indices were analyzed from the change curves. As shown in Fig 6, the influence of tunnel environment on the HR growth rate in the exit zone is pronounced 150 m before and 300 m after the exit. The HR growth rate shows a trend of slow decline, rapid increase, and gradual decline. However, the HR growth rate increases far more steeply and declines less with increasing tunnel length. The HR growth rate peaks at 100 m to 150 m after the tunnel (12.224%–20.345%). The decline in the HR growth rate is negatively correlated with the tunnel length. The RMSSD is mainly affected within the range of 300 m before to 200 m after the exit. The RMSSD reaches a peak 150 m before the exit and then declines to a nadir 25 m after the exit and then rises slightly. The RMSSD shows a positive correlation with the tunnel length. Before exiting the tunnel, the pupil diameter growth rate fluctuates by approximately 20% (17.12%–24.64%) and suddenly drops 50 m before the exit and gradually stabilizes after exiting the tunnel. The LP maintains a low until 50 m after the exit. The LP increases first to reach the highest point 150 m outside the tunnel and then decreases to level off.

### 3.3. Correlation between physiological indices and driving performance in tunnel entrance and exit zones

A comparative analysis was conducted between the physiological indices and LP in the entrance section. As shown in Fig 5, the change rules of the HR growth rate, RMSSD, and LP are relatively consistent. However, the variation in the HR growth rate appears later than that in the LP at the entrance, whereas the variation in the HR growth rate appears earlier than that in the LP at the exit. It can be speculated that the tunnel environment first affects the driving performance, and the changes in the environment and driving operations further lead to psychological tension. After entering the tunnel, the drivers first notice the changes in the driving environment in the tunnel, such as illumination and tunnel wall, resulting in a mental stress. By contrast, drivers are more cautious when entering the tunnel while decreasing the LP, so the change in the LP appears later than that in the HR. The HR growth rate and LP tend to stabilize after adapting to the environment. The variation rule of the pupil diameter is different from those of the HR growth rate, RMSSD, and LP. This may be attributed to the black hole effect produced by the considerable difference in illumination between the inside and outside of the tunnel, which sharply changes the pupil diameter and increases the load. However, the pupil diameter decreases gradually, and the load decreases to a certain extent as the driver slowly adapts to this phenomenon.

For the exit section, Fig 6 shows that the variation in the HR growth rate is relatively similar to that in the RMSSD, but differs from that in the LP. The HR indices are more sensitive to the changes in the driving environment at the tunnel exits. When approaching the exit, the drivers feel relaxed at first and then adapt to the light. The drivers spend more time driving in the dark and boring environment of longer tunnels, thus increasing the stress level and impairing the driving performance. The HR and LP stabilize after exiting the tunnel. The variation rule of the pupil diameter is different from those of the HR growth rate, RMSSD, and LP. This may be attributed to the white hole effect produced by the considerable difference in illumination between the inside and outside of the tunnel, thus causing a dramatic change in the pupil diameter. The pupil diameter finally levels off after the driver adapts to the environment outside the tunnel.

The main distinctions of the indices between the entrance and exit sections are as follows:

1. Drivers experienced two large fluctuations in the entrance section, mainly in the ranges of [−250 m, −75 m] and [0 m, 200 m]. By contrast, the drivers only experienced a significant fluctuation near the exit of the tunnel, mainly in the range of [−50 m, 200 m]. This indicates that the tunnel entrance section has a greater range of influence on the physiological response and driving performance of the drivers.
2. Drivers were more cautious at the tunnel entrance, as they drove while minimizing the LP. Therefore, the LP decreased first and then increased. The LP increased sharply 50 m after the tunnel exit, but peaked at approximately 150 m and then stabilized 225 m after the exit.
3. The change amplitudes of the indices were greater in the exit section than in the entrance section. This indicates that although the range of influence is greater at the entrance, the physiological changes due to the relief in mood are higher.
4. The growth rate of the pupil diameter shows a sudden drop while exiting the tunnel with a 20% reduction within 75 m. By comparison, the growth rate of the pupil diameter at the entrance exhibits a steady improvement. This could be attributed to the fact that a driver feels relaxed while exiting the tunnel but then experiences light adaption.
5. A correlation analysis showed that the LP is moderately correlated with the HR growth rate and RMSSD in the entrance section. In particular, the LP is positively correlated with the HR growth rate with a correlation coefficient (r) greater than 0.6 and an average correlation coefficient of 0.678. The LP is negatively correlated with the RMSSD (r = −0.652). However, the LP has little correlation with the physiological indicators of the drivers in the exit section, whereas the pupil diameter is positively correlated with the RMSSD (r = 0.727).

## 4. Discussion

In this study, the physiological indices and driving performance of drivers at tunnel entrances and exits in 12 tunnels with different lengths (1000–20000 m) were investigated through driving simulation experiments. The spatial variations in the HR growth rate, RMSSD, pupil diameter growth rate and LP of vehicles were analyzed to determine tunnel sections with a significant influence on the drivers. The relationship between the physiological indicators and the driving performance was further explored. Finally, the difference in the drivers’ physiology and driving performance between the exit and entrance sections is clarified.

The tunnel length had no significant influence on the HR growth rate, RMSSD, pupil diameter growth rate, and LP in the entrance section. It could be speculated that the main factor affecting the driver is the illumination variation. However, the HR growth rate and RMSSD showed marked differences between each tunnel in the exit section. It could be speculated that the long-time driving can increase drivers’ stress level, thus fluctuating the HR. Moreover, the HR growth rate, RMSSD, and LP were found to have two large fluctuations in the entrance section whereas only one significant fluctuation near the exit of the tunnel. This indicates that the black hole and white hole effects have different influences, with the black hole effect having a greater impact on the drivers. The variation pattern of the pupil diameter was significantly different from those of the HR growth rate, RMSSD, and LP. This is because the pupil diameter is mainly influenced by the light and brightness. In addition, the drivers tended to drive on the left side before entering the tunnel. This can be contributed to the fact that vehicles in China are driven on the right side with the rudder on the left. Affected by the tunnel wall, the drivers exhibit a left-driving tendency.

The physiological indices and driving performance of drivers at tunnel entrances and exits were analyzed in this study. The change in the physiological indicators represents the stress response of the drivers under the influence of the tunnel environment. The influence can lead to positive or negative effects. The positive effects include warming-up effect, activation and learning, whereas the negative effects can be mental fatigue and stress response [41]. It is typically impossible to directly evaluate whether the impact of physiological changes is positive or negative based on the value of the physiological indicators; nevertheless, this can be reflected indirectly by the driving performance. The driving performance is directly related to driving safety. For the tunnel sections, the main driving task is lane-keeping, which is restricted by the speed limit and tunnel environment. Therefore, this study adopted the LP of the vehicles to represent the driving performance. The inadequate lateral control of vehicle has been judged to reduce safety [42–44]. In this study, for the tunnel entrance section, the HR growth rate and RMSSD fluctuated significantly in the section with large LP fluctuation, indicating that such physiological changes had certain effects on the drivers. In the exit section, the LP had a large fluctuation after exiting the tunnel. However, in this section, the physiological indicators of the drivers remained steady except for the HR growth rate. It can be speculated that the changes in the driving behavior may be more influenced by the driving environment than by the physiological changes. Despite the fluctuation in the LP, the maximum value was approximately 45 cm, so the driving performance was relatively stable and within the controllable range. Our research illustrated the impacts of tunnel entrance and exit on a driver’s physiological indices and driving performance. The HR growth rate, RMSSD, and pupil diameter were found to fluctuate sharply at the tunnel portals, consistent with previous studies [5,7,10,15,17,20]. The tunnel entrance and exit significantly influence the driving performance. The LP fluctuates within the range of the entrance and exit, but is controllable. These findings are consistent with formal studies [3,20]. In addition, the tunnel entrance had a greater influence on the drivers than the exit section, and the drivers tried their best to keep their vehicles stable at the tunnel entrance by reducing the LP of the vehicles. A similar result can be found in [5]. In this study, the pupil diameter was found to increase quickly 100 m before the tunnel entrance. Similarly, the average fixation time increased and the number of fixations decreased 100 m before the tunnel entrance, indicating a heavy visual load [17,20]. Previous studies have shown that the accident rate is relatively high in a certain range (100–250 m) of the entrance and exit of tunnels. This study found that within this range, the HR, eye movement and vehicle LP vary significantly. Therefore, it could be speculated that the psychological pressure on the drivers is high in this section, which may affect their driving performance and impair the traffic safety. We concentrated on the impact of tunnels on the physiological indices and driving performance in a bidirectional four-lane expressway with an 80 km/h speed limit. The traffic flow and weather conditions affect drivers; however, we mainly explored the physiological characteristics at the tunnel entrance and exit sections. Therefore, traffic flow and environmental conditions are treated consistently. In the future, a comprehensive study considering other factors (speed limit, highway grade, and travel environment) on the physiological indices and driving performance can be conducted.

## 5. Conclusions

In this study, a simulation driving experiment was conducted to explore the physiological response and driving performance of drivers at the tunnel entrance and exit. The HR growth rate, RMSSD, and pupil diameter growth rate were chosen as the physiological indices. The LP of the vehicle was used to evaluate the driving performance. The spatial variations in the physiological indices and driving performance were analyzed, and the corresponding variation laws were obtained.

The tunnel entrance and exit had a significant impact on the physiological response and driving performance of the drivers. The HR growth rate increased sharply, and the RMSSD decreased gradually 250 m before the entrance. The pupil diameter increased considerably 100 m before the entrance. The LP had an evident fluctuation before the entrance, and the drivers tended to deviate to the left. Fifty meters before the exit of the tunnel, the driver’s HR first increased and then decreased, whereas the RMSSD first decreased and then increased, gradually recovering 200 m outside the tunnel. There were significant fluctuations in the LP and growth rate of the pupil diameter in the exit section. Moreover, the HR growth rate and RMSSD were significantly different at the exit of tunnels with different lengths. The greater the tunnel length, the greater the variation range of the index. The tunnel length had no significant effect on the physiological indicators and driving performance of the drivers. The entrance section had a greater influence on the drivers than the exit section. The research findings are of great significance for the design and management of tunnels in expressway. First, the results of this study can help advance our understanding of the mechanism of tunnel accidents, thus guiding the development of tunnel management policies. Second, effective traffic signs should be set up at least 250 m before the entrance. The sign information should be simple and easy to recognize, in order to help prepare the drivers while entering the tunnel in advance and drive prudently. Moreover, appropriate guides can be set to remind the drivers of the following driving environment at least 50 m before leaving the tunnel, thus relieving the pressure in the tunnel environment. Overall, the findings of this paper provide a reference for the rational design and effective management of road systems in the context of improving driving safety.

## Supporting information

S1 Text A blank copy of informed consent. (PDF)

S2 Text Certification of the ethical review for the experiment. (PDF)
