# Supplementary material for: Physiological indices and driving performance of drivers at tunnel entrances and exits: A simulated driving study
Source: PLoS One. 2020 Dec 17;15(12):e0243931. doi: 10.1371/journal.pone.0243931 (PMC7746149; doi:10.1371/journal.pone.0243931)
Supplement: S1 Text — (PDF) [file pone.0243931.s001.pdf]

《高速公路道路设施和几何特性对驾驶员工作负荷与驾驶行为影响》研究

受试者知情同意书

尊敬的受试者：

您将参加一项科研实验。本须知提供给您一些信息以帮助您决定是否参与此次实验。请您仔细阅读，如有疑问请向负责本实验的研究者提出。

您参与的本项实验是自愿的。本次研究符合《赫尔辛基宣言》的原则。

实验目的：分析驾驶员在高速公路不同路段行驶时的工作负荷和驾驶行为表现。

实验对象：身体健康、视力 5.0 以上、有驾驶执照且驾龄在 5 年以上的驾驶员。

实验过程：如果您同意参与这项实验，我们将对您的锁骨下方、右侧腹部和正腹部用酒精清洗后再用医用电极，同时佩戴 SMI 型眼动仪。在实验过程中，您将需要静坐在驾驶模拟舱内，根据驾驶经验依次完成本实验，在此过程中我们将通过 MP150 生理仪和 SMI 眼动仪收集您的心电数据和眼动数据，此实验时长约 5 个小时（包含实验中间的休息时间）。

风险与不适：采集数据所用的电极均已消毒，且导电膏对皮肤无副作用。数据采集过程中，SMI 眼动仪对眼睛无影响。如有不适，受试者可随时提出终止实验。

您可随时了解与本研究有关的信息资料和研究进展，如果您有与本研究相关的问题，或在实验过程中发生了任何不适与损伤，或与关于本研究参加者权益方面的问题，您可以通过电话 18792896968 与实验员张晓冬联系。

受试者声明：

我已阅读了本知情同意书，且已与本实验的研究者详细讨论并了解本研究的目的、对象、过程和风险。在仔细阅读以上有关说明后，经过充分时间的考虑，我自愿成为此项研究的受试者，积极配合研究人员进行本项实验。

受试者签名：

联系电话：　　　　　　　　　　　　　　　　　　　日期：

研究者签名：

联系电话：　　　　　　　　　　　　　　　　　　　日期：
